# Low-Dose Methylmercury-Induced Genes Regulate Mitochondrial Biogenesis via miR-25 in Immortalized Human Embryonic Neural Progenitor Cells

**DOI:** 10.3390/ijms17122058

**Published:** 2016-12-09

**Authors:** Xinjin Wang, Mengling Yan, Lina Zhao, Qing Wu, Chunhua Wu, Xiuli Chang, Zhijun Zhou

**Affiliations:** School of Public Health and Key Laboratory of Public Health Safety of the Ministry of Education, Fudan University, Shanghai 200032, China; fjwangxinjin@163.com (X.W.); 13211020073@fudan.edu.cn (M.Y.); 14211020022@fudan.edu.cn (L.Z.); qingwu@fudan.edu.cn (Q.W.); chwu@shmu.edu.cn (C.W.); zjzhou@fudan.edu.cn (Z.Z.)

**Keywords:** methylmercury, mitochondria biogenesis, microRNA, developmental neurotoxicity

## Abstract

Mitochondria are essential organelles and important targets for environmental pollutants. The detection of mitochondrial biogenesis and generation of reactive oxygen species (ROS) and p53 levels following low-dose methylmercury (MeHg) exposure could expand our understanding of underlying mechanisms. Here, the sensitivity of immortalized human neural progenitor cells (ihNPCs) upon exposure to MeHg was investigated. We found that MeHg altered cell viability and the number of 5-ethynyl-2′-deoxyuridine (EdU)-positive cells. We also observed that low-dose MeHg exposure increased the mRNA expression of cell cycle regulators. We observed that MeHg induced ROS production in a dose-dependent manner. In addition, mRNA levels of peroxisome-proliferator-activated receptor gammacoactivator-1α (PGC-1α), mitochondrial transcription factor A (TFAM) and p53-controlled ribonucleotide reductase (p53R2) were significantly elevated, which were correlated with the increase of mitochondrial DNA (mtDNA) copy number at a concentration as low as 10 nM. Moreover, we examined the expression of microRNAs (miRNAs) known as regulatory miRNAs of p53 (i.e., miR-30d, miR-1285, miR-25). We found that the expression of these miRNAs was significantly downregulated upon MeHg treatment. Furthermore, the overexpression of miR-25 resulted in significantly reducted p53 protein levels and decreased mRNA expression of genes involved in mitochondrial biogenesis regulation. Taken together, these results demonstrated that MeHg could induce developmental neurotoxicity in ihNPCs through altering mitochondrial functions and the expression of miRNA.

## 1. Introduction

Methylmercury (MeHg) is a ubiquitous environmental toxicant. Because MeHg can be bioaccumulated in aquatic food chain, chronic exposure to MeHg occurs mainly via consumption of large amounts of fish and shellfish in human beings [1,2]. Furthermore, MeHg can readily cross biological membranes such as the blood–brain barriers and the placenta, and the concentration in umbilical cord blood can be substantially higher than in maternal blood [3,4]. These observations have led to a wide range of investigations to identify the detrimental effects of MeHg on the adult and developing central nervous system. Indeed, epidemiological studies have shown that exposure to much lower levels of MeHg is correlated with general disturbances of cognitive thinking, memory, attention, language, and fine motor and visual spatial skills [5,6] though several studies have produced ambiguous, often contradictory results [7]. Similar results have been reported in animal models of brain development after exposure to MeHg in utero [8,9]. Moreover, extensive evidence has demonstrated that MeHg can lead to neural cell death, as well as to cytoarchitectural alterations in the nervous system [10,11,12,13,14]. There is an abundance of knowledge regarding reactive oxygen species (ROS) and especially effects of low-level MeHg exposure on cell cycle regulators in neural stem cells in vitro and in vivo [15,16,17]. However, effects on function in human neural stem cells are still in unknown, especially in immortalized human neural progenitor cells (ihNPCs).

Mitochondria are critical for normal cell and organ function. Maintenance of mitochondrial DNA (mtDNA) integrity and copy number is fundamental to sustaining mitochondrial function. The amount of cellular mitochondria and their contents are regulated via mitochondrial biogenesis, nuclear signaling-mediated nuclear and mitochondrial transcription, autophagy, and intraorganellar degradation processes. A leading hypothesis has been reported that MeHg-induced neurotoxicity is due to the generation of ROS in stressed mitochondrial respiration chain [17,18]. Increased oxidative stress may contribute to alterations in the abundance of mitochondria as well as in the copy number and integrity of mtDNA in human cells in pathological conditions and in the aging process. Moreover, accumulating evidence indicates that the oxidative stress induced by MeHg can increase the expression of p53 [19] which works as a guardian of the mitochondria genome [20]. Specifically, p53 plays an essential role in the transcriptional regulation of several genes involved in maintaining mitochondrial functions, including nuclear DNA-encoded mitochondrial transcription factor A (TFAM), encoding p53-controlled ribonucleotide reductase (p53R2) and peroxisome-proliferator-activated receptor gammacoactivator-1α (PGC-1α) [21]. However, the potential role of disturbed mitochondrial biogenesis and associated oxidative stress as well as impaired neural progenitor proliferation induced by MeHg exposure in ihNPCs, especially at very low levels of MeHg, remain unknown.

MicroRNAs (miRNAs) are a recently recognized group of small, endogenous non-coding RNAs that can downregulate gene expression by interfering with mRNA functions. They play critical roles in mammalian development, especially in neuronal development, from early neurogenesis to neuronal differentiation and synaptic development, and also in in vitro systems [22,23]. However, the role of miRNAs in MeHg-induced neurotoxicity remain poorly understood.

In the current study, we investigated the effect of low-dose MeHg on mitochondrial biogenesis in ihNPCs, which has been widely used as an in vitro model for the study of nervous system development [24]. We elucidate the underlying mechanisms of mitochondrial dysfunction induced by low-dose MeHg along with ROS generation and p53 upregulation. In addition, we have determined that MeHg can modulate mitochondrial biogenesis and alter mitochondrial functions in ihNPCs. Furthermore, we have determined that MeHg can alter the expression of miRNAs which are important regulators of p53.

## 2. Results

### 2.1. Immortalized Human Neural Progenitor Cells Are Highly Susceptible to MeHg Toxicity

**Figure 1:**
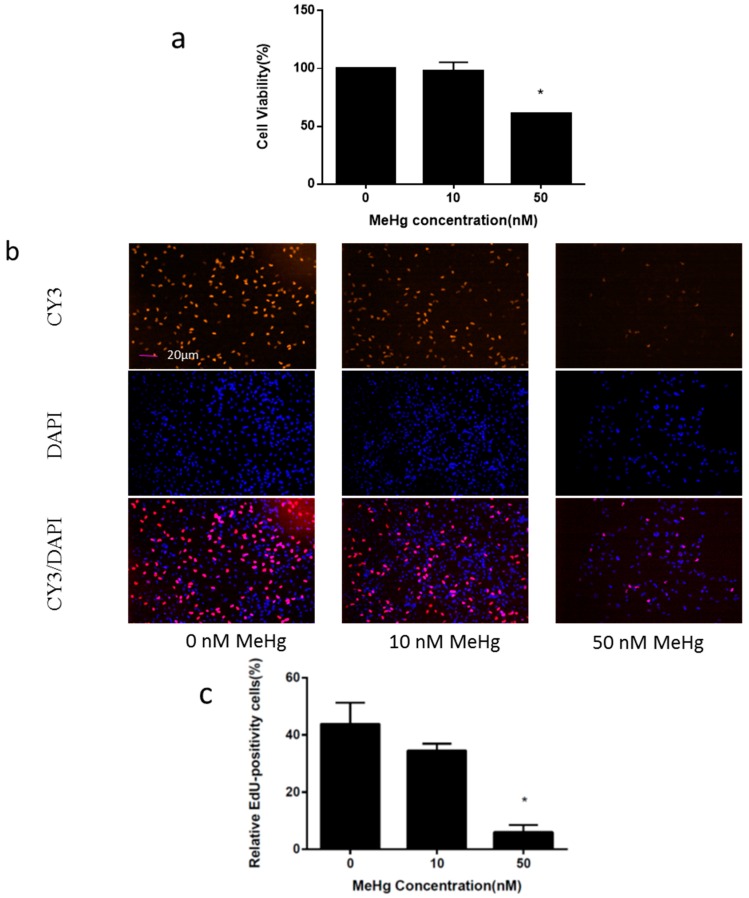
Immortalized human neural progenitor cells (ihNPCs) were highly susceptible to methylmercury (MeHg)-induced toxicity. (**a**) Altered cell viability induced by MeHg treatment; (**b**) 5-Ethynyl-2′-deoxyuridine (EdU)-positive cells induced by MeHg treatment. Fluorescent image of EdU incorporation and staining in ihNPCs after MeHg treatment. Blue: 4′,6-diamidino-2-phenylindole (DAPI); Red: cyanine (CY) 3. Scale bar: 20 µm; (**c**) Quantification of Edu-positive cells. Results were expressed as mean ± SEM (*n* = 3). * *p* < 0.05 when compared with the corresponding control group (0 nM MeHg).

The ihNPCs were exposed to MeHg at the concentrations 0 nM, 10 nM and 50 nM for 24 h. The effect of MeHg exposure on the viability of ihNPCs was determined by Cell Counting Kit-8 (CCK-8) assay. We observed that cell viability was not affected at 10 nM of MeHg, but was significantly reduced to 61% at 50 nM MeHg treatments (Figure 1a). Next, we determined the ihNPC proliferation upon MeHg treatment using 5-ethynyl-2′-deoxyuridine (EdU), which serves as a cell proliferation marker incorporated in cells during the S-phase of the cell cycle. In the following experiments, MeHg was found to inhibit the number of EdU-positive cells in ihNPCs in a dose-dependent manner, and a significant inhibitory effect was observed at a concentration of 50 nM (Figure 1b,c).

### 2.2. Effect of MeHg on Cell Cycle Regulatory Genes

**Figure 2:**
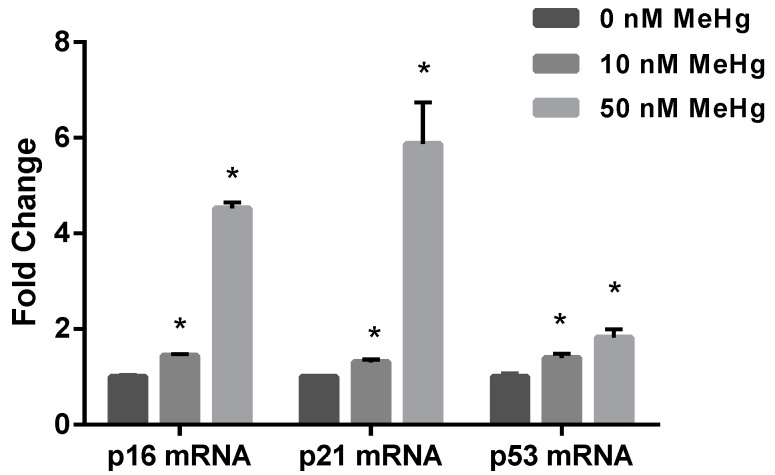
Effect of MeHg on cell cycle regulatory genes in ihNPCs. Quantitative real time polymerase chain reaction (qPCR) analysis of p16, p21 and p53 mRNA expression levels performed on RNA extracted from ihNPCs treated with 0 nM, 10 nM and 50 nM MeHg, respectively. β-Actin was used for normalization. Fold change RNA was normalized over 0 nM MeHg. Results are expressed as mean ± SEM (*n* = 3). * *p* < 0.05 when compared with the corresponding control group (0 nM MeHg).

In this study, we examined the mRNA expression levels of p16, p21 and p53 to understand their roles in MeHg-induced cell damage. Quantitative real time polymerase chain reaction (qPCR) analysis revealed a significant MeHg-induced upregulation of p16, p21 and p53 mRNA expressions (p16 fold change: 10 nM, 1.44 ± 0.04; 50 nM, 4.52 ± 0.13; p21 fold change: 10 nM, 1.31 ± 0.06; 50 nM, 5.87 ± 0.87; p53 fold change: 10 nM, 1.40 ± 0.09; 50 nM, 1.82 ± 0.17; Figure 2).

### 2.3. Effect of MeHg on Intracellular Reactive Oxygen Species Generation

**Figure 3:**
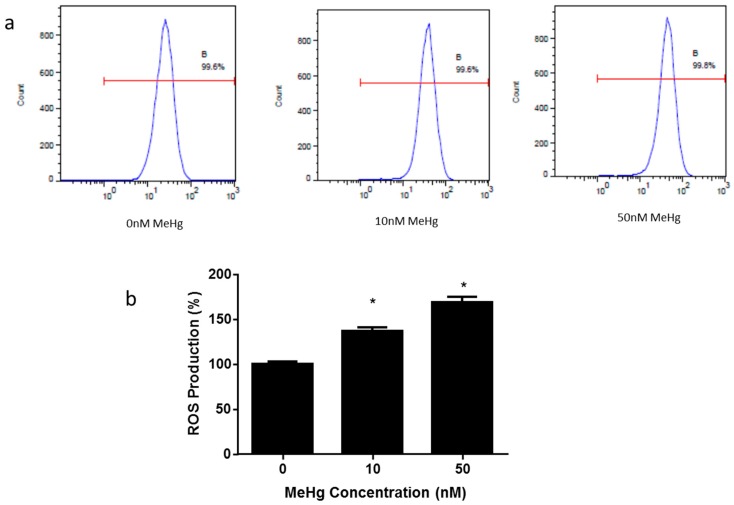
Effect of MeHg on the production of reactive oxygen species (ROS) in ihNPCs. Cells were treated with 0 nM, 10 nM, and 50 nM MeHg, and the level of ROS was analyzed. (**a**) ROS levels after MeHg treatment were analyzed by flow cytometry; (**b**) The relative ROS levels were presented as fold differences based on those at 0 nM. Results are expressed as mean ± SEM (*n* = 3). * *p* < 0.05 when compared with the corresponding control group (0 nM MeHg).

Much evidence has shown that oxidative stress represents a critical event related to MeHg-induced neurotoxicity both in vivo and in vitro [17]. In this study, the effect of MeHg on the generation of ROS was measured by flow cytometry (Figure 3a). ROS production was significantly increased in 10 nM and 50 nM MeHg-treated cells (10 nM, 1.47 ± 0.05; 50 nM, 1.69 ± 0.06), in comparison to control cells (Figure 3b).

### 2.4. MeHg Alters Genes Regulating Mitochondrial Biogenesis

**Figure 4:**
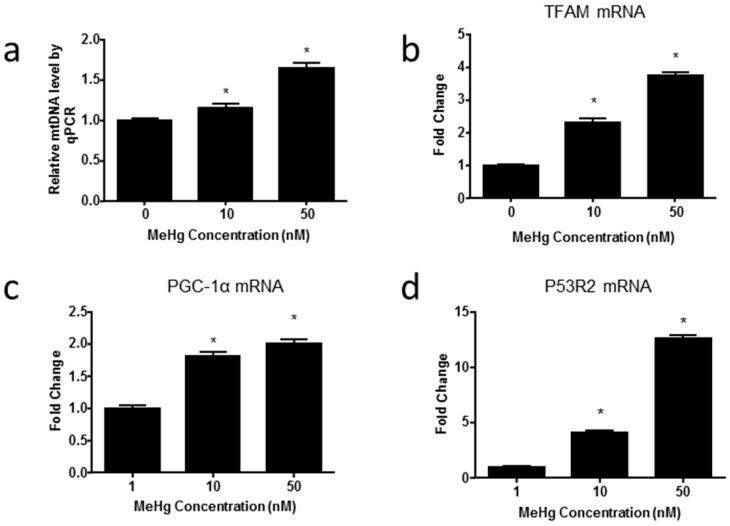
MeHg alters the expression of genes regulating mitochondrial biogenesis (**a**) The relative mitochondrial DNA (mtDNA) were presented as fold differences based on control. Results are expressed as mean ± SEM (*n* = 3). * *p* < 0.05 when compared with the corresponding control group (0 nM MeHg); (**b**–**d**) qPCR analysis of peroxisome-proliferator-activated receptor gammacoactivator-1α (PGC-1α), mitochondrial transcription factor A (TFAM) and p53-controlled ribonucleotide reductase (p53R2) mRNA expression levels performed on RNA extracted from ihNPCs treated with 0 nM, 10 nM and 50 nM of MeHg. Relative mRNA levels were presented as fold differences based on control. Results are expressed as mean ± SEM (*n* = 3). * *p* < 0.05 when compared with the corresponding control group (0 nM MeHg).

To investigate whether MeHg can induce changes in the amount of mtDNA, we first examined the relative mtDNA copy number in ihNPCs by normalizing mitochondrially encoded nicotinamide adenine dinucleotide (NADH):ubiquinone oxidoreductase core subunit 1 (*MT-ND1*) gene to a single-copy number human β-globin (*hbg*) gene (Figure 4a). The mtDNA *MT-ND1* gene encodes the NADH dehydrogenase subunit of the electron transport chain (ETC) complex I and we used it as a measure of the mtDNA copy number. We observed that MeHg led to a significant increase of the mtDNA content in ihNPCs (fold change: 10 nM, 1.14 ± 0.06; 50 nM, 1.65 ± 0.06). Then, we investigated the expressions of several regulators of mitochondrial biogenesis by qPCR, and we found that the expressions of PGC-1α, TFAM and p53R2 were significantly increased after MeHg treatment, which were correlated with the increase of mtDNA copy number (PGC-1α fold change: 10 nM, 1.81 ± 0.06; 50 nM, 2.00 ± 0.07; TFAM fold change: 10 nM, 2.30 ± 0.12; 50 nM, 3.74 ± 0.10; p53R2 fold change: 10 nM, 4.09 ± 0.18; 50 nM, 12.59 ± 0.32) (Figure 4b–d).

### 2.5. Low-Level MeHg Induces Genes Regulating Mitochondrial Biogenesis via miR-25

**Figure 5:**
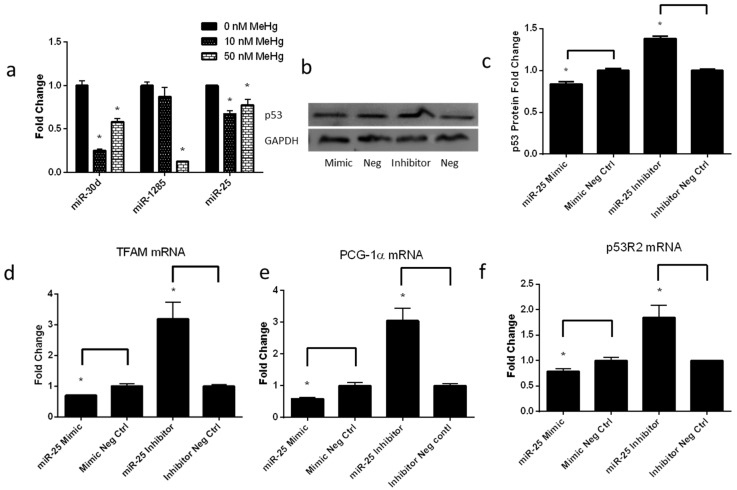
Low-level MeHg induces genes regulating mitochondrial biogenesis via miR-25. (**a**) qPCR analysis of mature miR-30d, miR-1285, and miR-25 expression levels performed on RNA extracted from ihNPCs treated with 0 nM, 10 nM and 50 nM MeHg; (**b**) A representative Western blot of p53 expression in ihNPCs. Cells were transfected with 50 nM of miR-25 mimics, miR-mimic negative control#1, miR-25 inhibitor and miR-inhibitor negative control#1 for 48 h; (**c**) Western blot analysis of p53 expression performed in 10 nM-treated ihNPCs after transient transfection of miR-25 mimics, miR-mimic negative control#1, miR-25 inhibitor and miR-inhibitor negative control#1. Glyceraldehyde 3-phosphate dehydrogenase (GAPDH) protein expression has been used as a loading control and a representative figure of three independent experiments has been shown. Results of gray value analysis are also reported (left part); (**d**–**f**) qPCR analysis of TFAM, PCG-α and p53R2 expression performed in 10 nM-treated ihNPCs after transient transfection of miR-25 mimics, miR-mimic negative control#1, miR-25 inhibitor and miR-inhibitor negative control#1. All experiments were repeated three times (technical triplicates) with biological triplicates (*n* = 3). Bar graphs show mean ± SEM (* *p* < 0.05).

As a mito-checkpoint protein, p53 plays an important role in regulating mtDNA copy number and genes regulating mitochondrial biogenesis [20]. In addition, we recently discovered that p53 was targeted by miR-1285, miR-25 and miR-30d through analysis using both bioinformatics tools (miRGen, TargetScan, Pictar, and Miranda) and the most current literature [25,26,27]. Therefore, we examined the changes in these miRNAs (miR-30d, miR-1285, miR-25), which may have a potential impact on p53 expression. As shown in Figure 5a, qPCR analysis revealed a significant MeHg-induced downregulation of the three miRNAs in ihNPCs (miR-30d fold change: 10 nM, 0.25 ± 0.02; 50 nM, 0.58 ± 0.04; miR-1285 fold change: 10 nM, 0.87 ± 0.11; 50 nM, 0.13 ± 0.01; miR-25 fold change: 10 nM, 0.68 ± 0.03; 50 nM, 0.78 ± 0.07).

To investigate the role of miRNA in regulating p53 expression and genes regulating mitochondrial biogenesis, we transfected the miR-25 mimics, the miR-mimic negative control#1, the miR-25 inhibitor and the miR-inhibitor negative control#1 into 10 nM MeHg-treated ihNPCs, and examined the p53 protein levels by Western blot. We found that ectopic miR-25 overexpression leads to ~20% reduction in p53 protein levels. On the contrary, anti-miR-25 treatment led to an upregulation of p53 level, in which a 40% increase in p53 protein levels was observed (Figure 5b,c). In addition, the mRNA expression of markers in mitochondrial biogenesis, such as PGC-1α, TFAM and p53R2, were significantly reduced in ihNPCs transfected with the miR-25 mimics. On the contrary, anti-miR-25 treatment strongly enhanced the mRNA expression of PGC-1α, TFAM and p53R2 (Figure 5d–f). Collectively, these results demonstrated that MeHg might tune p53 expression and influence mitochondrial biogenesis via miRNAs.

## 3. Discussion

In this study, we demonstrated that exposure to nanomolar concentrations of MeHg (10 nM), which failed to induce the S-phase arrest displayed by EdU-positive ihNPCs, can increase generation of ROS and promote genes regulating mitochondrial biogenesis in ihNPCs. In addition, we showed that exposure to 50 nM MeHg can reduce the number of EdU-positive ihNPCs and viability, which is associated with changes of gene expressions of cell cycle regulators. Moreover, our results suggested that MeHg-induced genes regulating mitochondrial biogenesis in ihNPCs may be regulated by p53 via miRNAs.

We have selected ReNcell CX cells, which are immortalized human neural progenitor cells possessing self-renewal and multipotent differentiation capacity, for use as an in vitro developmental neurotoxicity model to investigate the cytotoxic effects of low doses of MeHg. Previous studies [24,28] demonstrated that ReNcell CX cells were an appropriate in vitro model of ihNPCs for developmental neurotoxicity induced by a set of compounds including MeHg. In this study, to mimic the environmental exposure, we used low doses of MeHg (10 nM and 50 nM), which are below the dose levels used in other cell-based models [29]. We found that 24 h exposure to low doses of MeHg (50 nM) inhibited the number of EdU-positive cells and viability of cultured ihNPCs (Figure 1). Furthermore, there were significant decreases in cell numbers due to the inhibition of ihNPC DNA synthesis. Consistently, Burke et al. have demonstrated that cell cycle arrest was an early target of MeHg toxicity, and proposed that cyclin E degradation contributed to reduced proliferation altered cell cycle and apoptosis in a rat model exposed to MeHg [15].

Our study revealed that MeHg significantly increased cell cycle regulatory molecules, such as mRNA expressions of p16, p21 and p53 in ihNPCs (Figure 2c). Importantly, it has been shown that tumor suppressor protein p53 participates in multiple cellular processes, such as cell cycle arrest, senescence, and apoptosis [30]. Furthermore, p53 has an intricate relationship with ROS generation. For example, hyper- and physiological levels of ROS can transactivate a series of p53-induced genes and these genes are critical in regulating ROS production and in turn are likely to determine cellular redox homeostasis [31]. It has been reported that MeHg can enhance the level of ROS in both in vivo and in vitro models [10]. Depending on their concentrations, ROS can have either protective or harmful effects on tissues. At moderate levels, ROS function as regulatory mediators to modulate genes involved in signaling processes. However, excessive amounts of ROS would cause oxidative injury which has been implicated in various diseases [32,33,34]. Our data demonstrated that low-dose MeHg can cause an increase in the generation of ROS in ihNPCs in a dose-dependent manner (Figure 3), which was consistent with the changes of p53 mRNA expression (Figure 2c). These results are in agreement with previous findings [35,36].

Mitochondria are believed to be the major targets of MeHg-induced toxicity [37], and a primary source of intracellular ROS. In the current study, we observed that MeHg-treated ihNPCs had increased ROS production, which was associated with elevated mtDNA copy number. In contrast, mRNA expression of regulatory genes involved in mitochondrial biogenesis, such as PGC-1α, TFAM and p53R2 (Figure 4), failed to lead to cell cycle arrest, as an early event of MeHg-induced toxicity [15]. Presumably, these alterations may be due to the increases in mtDNA copy number which has been suggested to work as a feedback response to compensate for defective mitochondria bearing impaired respiratory chain. In turn, an increase in mtDNA contents could contribute to further oxidative stress leading to damaged mtDNA, mitochondrial dysfunction, oxidative damage and stem cell aging [38,39]. Noteworthy, it has been reported that increases of mitochondrial biogenesis are not always beneficial to cells, and can elicit senescent of stem cell [40]. Consistently, our results showed that levels of the senescence maker p16 were increased at a dose of 10 nM (Figure 2c), although these effects could represent the first response to MeHg. Similarly, the previous study showed that MeHg had no adverse effect on cell viability but altered the expression of senescence-associated markers [16]. The results observed in the experimental model suggest that very low-dose MeHg exposure induced premature senescence of stem cells, which may be associated with increases of mitochondrial biogenesis. Further research is needed about the mechanisms.

There is increasing evidence showing direct actions of p53 and ROS at the mitochondria. For example, they not only to some degree induce mitochondrial apoptotic changes, but also influence mitochondrial biogenesis as well as the copy number and integrity of mtDNA in human cells [21,38]. Saleem et al. reported that p53 knockout mice had reduced mitochondrial content and decreased expressions of PGC-1α, but elevated productions of ROS in skeletal muscle compared to wild-type mice [41]. p53 could protect mtDNA integrate through p53R2 [20]. p53 could also regulate the replication, transcription and repair of mtDNA through modulating the expression of PGC-1α and other transcription factors, such as nuclear respiratory factor 1 (NRF-1), NRF-2 and TFAM which act on the promoters within the D-loop region (noncoding region) of mtDNA and regulate its replication, transcription and repair [42]. In addition, mtDNA lacking protective histones and efficient repair mechanisms, are vulnerable to damage by excessive ROS [43]. Furthermore, an increasing number of studies have focused on demonstrating the important role of miRNAs in the regulation of p53 expression. In this study, we investigated the changes in explicit miRNAs miR-1285, miR-30d and miR-25 which have been recognized as regulators of p53 [25,26,27]. We observed that these miRNAs were significantly repressed when ihNPCs were exposed to MeHg (Figure 5a). To explore the functional significance of these miRNAs in MeHg-induced p53 expression, we chose miR-25 for further investigation. Moreover, we checked miR-25 target genes through Mirwalk (a database which presents predicted and validated information on miRNA-target interaction [44]) and found that miR-25 may not directly regulate the expression of PGC-1α, TFAM and p53R2. Interestingly, we found that miR-25 overexpression significantly reduced the protein expression of p53 in MeHg-treated ihNPCs. However, suppression of miR-25 expression dramatically increased p53 protein levels. We also found that miR-25 overexpression inhibited mtDNA biogenesis gene (PGC-1α, TFAM and p53R2) expression. Taken together, our results suggest that low-dose MeHg-induced mitochondrial biogenesis is influenced by p53 via miR-25 in ihNPCs. Although there are discoveries revealed by these studies, there are also limitations. Firstly, the diverse effects associated with MeHg-induced developmental neurotoxicity cannot be explained as a single process. Moreover, it should be noted that this study has examined only immortalized human neural progenitor cells not primary neural progenitor cells.

## 4. Material and Methods

### 4.1. Cell Culture

Immortalized human neural progenitor cells (ihNPCs, ReNcell CX cells) were obtained commercially from Millipore (Temecula, CA, USA). Cells were recovered and cultured on a 100 mm diameter dish pre-coated with laminin (Sigma-Aldrich, Milan, Italy), using ReNcell NSC maintenance medium (Millipore, Temecula, CA, USA) with fresh epidermal growth factor (EGF) (20 ng/mL; Millipore) and fibroblast growth factor 2 (FGF 2) (20 ng/mL; Millipore), as described previously [45].

### 4.2. MeHg Treatment

MeHg (Sigma-Aldrich) was dissolved in dimethyl sulfoxide (DMSO). ihNPCs were seeded at a density of 2.5 × 10^4^ per well in laminin-coated 96-well plates (Corning Inc., New York, NY, USA). After 24 h incubation, the culture medium was changed and MeHg (0 nM, 10 nM and 50 nM) was added and maintained for another 24 h. All experiments were performed in triplicates and repeated at least three times.

### 4.3. Assay of Cell Viability

The viability of ihNPCs was determined using the CCK-8 assay (Beyotime, Jiangsu, China) [46]. After MeHg treatment for 23 h, 10 μL CCK-8 solutions were added to each well, followed by incubation for 1 h at 37 °C. The optical density (OD) was measuredusing a Synergy HT microplate reader (Biotek Instruments, Inc., Winooski, VT, USA) at a test wavelength of 450 nm. The calculation of relative cell survival rate (cell viability, CV) is: CV = (OD of experimental group/OD of 0 nM group) × 100%.

### 4.4. 5-Ethynyl-2’-deoxyuridine Incorporation Assay

ihNPCs were seeded at a density of 2.5 × 10^4^ per well in laminin-coated 96-well plates (Corning, NY, USA). After 24 h incubation, the culture medium was changed and MeHg was then added at the concentration ranging from 0 nM, 10 nM and 50 nM, respectively, and cultured for 24 h. The effect of MeHg on cell proliferation was measured using Cell-Light EdU Apollo Kit (RiboBio, Guanzhou, China), as described previously [45]. The results were expressed as EdU+ cell numbers per 100 4′,6-diamidino-2-phenylindole (DAPI) (RiboBio, Guanzhou, China) stained cells.

### 4.5. Reactive Oxygen Species Measurement

Intracellular ROS production was measured using 2′,7′-dichlorodihydrofluorescein diacetate (DCF-DA; Molecular Probes, Beyotime, Jiangsu, China) [47]. The ihNPCs were grown to 80% confluence in 3 cm laminin-coated culture dish (Corning, Inc., Corning, NY, USA), then treated with MeHg (10 nM and 50 nM) for 24 h; positive control groups were treated with Rosup (Beyotime, Jiangsu, China) (an ROS stimulation standard) for 30 min at 37 °C in dark conditions; and then incubated with 100 µL of 1 × DCFH-DA/media solution placed at 37 °C for 30 min. The fluorescence intensity of the DCF-DA was measured using flow cytometry (Epics Altra, Beckman Coulter, Hialeah, FL, USA) with excitation and emission wavelength at 488 and 530 nm, respectively. The percentage increase of the ROS produced in the MeHg-treated cell was normalized over 0 nM values.

### 4.6. Analysis of mtDNA Copy Number by Quantitative Real-Time PCR

Relative mtDNA copy number was measured using a quantitative qPCR assay by determining the ratio of mitochondrial copy number to single (S) copy number in experimental samples. This method is based on the quantification of mtDNA and S quantities expressed as Cts derived from a standard curve obtained from serial dilutions of a reference DNA. The reference single copy gene used in this study was hbg.

Total cellular DNA was isolated using the QIAamp DNA Mini kit (Qiagen, Hilden, Germany) according to the manufacturer’s protocol. qPCR standards were generated by qPCR as described previously [48]. Briefly, 2 µg DNA was amplified in 50 µL reactions using 5 × PCR buffer (Bioline, Luckenwalde, Germany), 1.5 mM MgCl_2_ (Bioline), 100 mM dNTPs (Bioline), 0.5 mM for each of the forward and reverse primers. PCR products were resolved on 2% agarose gels at 100 V for 1 h, and then purified by gel-extraction kit (TIANgen Biotech, Beijing, China). A series of 10-fold dilutions (1 × 10^3^ ng/µL to 1 × 10^8^ ng/µL) of the target-specific PCR product was generated. The reference single copy gene used in this study was hbg. The mtDNA PCR mix included 1 µL template DNA, 5 µL SYBR Green PCR Master Mix (Applied Biosystems, Carlsbad, CA, USA), 3 µL ddH_2_O, 1 µL (200 nM) of forward and reverse primers mix at one cycle of 95 °C for 10 min, 40 cycles of 95 °C for 15 s, and 60 °C for 1 min on ABIStepone Plus Real Time PCR Detection System (Applied Biosystems). The primers (all primers were purchased from Sangon Biotech, Shanghai, China) for qPCR analysis of mtDNA were: MT-ND1-F, 5′-CCCTAAAACCCGCCACATCT-3′; MT-ND1-R, 5′-GAGCGATGGTGAGAGCTAAGGT-3′. The primers for hbg were: hbg-F, 5′-GCTTCTGACACAACTGTGTTCACTAGC-3′; hbg-R, 5′-CACCAACTTCATCCACGTTCACC-3′. Melting curve analysis was performed for each run to confirm the amplification specificity and absence of primer dimers, as described previously [49].

### 4.7. Quantitative Real Time PCR of mRNA and miRNAs

Total cellular RNA was isolated using Trizol reagent (Invitrogen, Carlsbad, CA, USA) according to the manufacturer’s recommendations, followed by a reverse transcription with cDNA synthesis kit (ThermoFisher, Rockford, IL, USA). cDNA was synthesized from 2 µg of total RNA using 1 µL of reverse transcriptase and 50 ng/mL oligo(dT).

For detection of mature miRNAs (miRNA-1285, miRNA-25 and miRNA-30d), 500 ng of total RNA, miRNA-specific stem–loop RT primers (RiboBio), and PrimeScript RT reagent Kit (Qiagen, Valencia, CA, USA) were used in reverse transcription. U6 small nuclear RNA (RNU6B) was used as an internal control to determine relative miRNA expression. Each qPCR was carried out in triplicate using SYBR Green PCR Master Mix (Applied Biosystems) at one cycle of 95 °C for 10 min, 40 cycles of 95 °C for 15 s, and 60 °C for 1 min on ABISteponePlus Real Time PCR Detection System (Applied Biosystems, USA). PCR mix was: 1 µL template cDNA, 5 µL SYBR Green PCR Master Mix (Applied Biosystems, USA), 3 µL ddH_2_O, 1 µL (200 nM) of forward and reverse primers mix. All expression values were normalized against the housekeeping gene β-actin or the U6 (Δ*C*_t_ = *C*_t target gene_ − *C*_t β-actin/U6_). Relative expression levels were then calculated as ΔΔ*C*_t_ = Δ*C*_t MeHg_ − Δ*C*_t control_, and relative expression changes were calculated as 2^−ΔΔ*C*t^. PCR primer sequences are available in the Table 1.

### 4.8. Cell Transfection

Cells were transiently transfected using the Lipofectamine 2000 reagent (Invitrogen) as recommended by the manufacturer. ihNPCs were transfected with 30 nM of miR-25 mimics, miR-mimic negative control#1, miR-25 inhibitor or miR-inhibitor negative control#1 (Invitrogen). Transfections were performed 24 h before MeHg treatment. After 48 h transfection, cells were harvested for appropriate subsequent assays.

### 4.9. Western Blotting

Western blots were performed as described previously [50]. Total proteins of ihNPCs were isolated using sodium dodecyl sulfate (SDS) sample buffer. Lysates were loaded by 10% sodium dodecyl sulfate–polyacrylamide gel electrophoresis (SDS-PAGE) and transferred to a PVDF membrane (Millipore, Bedford, MA, USA). The membrane was blocked with 5% skim milk for 1 h at room temperature and then incubated with primary antibodies to p53 and glyceraldehyde 3-phosphate dehydrogenase (GAPDH; Life Technology, Carlsbad, CA, USA) at 4 °C for 24 h. On the following day, the membrane was washed with 0.1% Tween-20 in Tris-buffered saline (TBS-T) and then incubated with horseradish peroxidase (HRP)-conjugated secondary antibody (anti-mouse) (Life Technology) for 1 h. The membrane was reacted with enhanced chemiluminescent (ECL) solution (ThermoFisher, Carlsbad, CA, USA), and LAS-3000 mini (Fujifilm, Tokyo, Japan) chemiluminescence detection device was used to visualize the labels. Band intensities were quantified by using a densitometer analysis system and expressed as integrated optical density (IOD). Target protein densitometry values were adjusted to GAPDH intensity, then normalized to expression from the control sample.

### 4.10. Statistical Analysis

Statistical analysis was performed using Stata 10.0 statistic program (StataCorp., College Station, TX, USA). Data are shown as mean ± SEM. Multiple group comparisons were carried out by one-way analysis of variance (one-way ANOVA), followed by Bonferroni’s post hoc test. A *p* < 0.05 was considered statistically significant.

## 5. Conclusions

Our study provides evidence that low-dose MeHg exposure accelerates mitochondrial respiratory chain-induced oxidative stress, elevating p53 expression, increasing mtDNA contents and promoting ROS generation in ihNPCs. Moreover, we found that MeHg can inhibit miR-25 expression levels in ihNPCs. Suppression of miR-25 expression dramatically increased p53 protein levels and mtDNA biogenesis gene expression. In addition, we demonstrated that MeHg-induced alterations in miRNA expression play potential roles in regulating p53 expression, which provide novel evidence for the function of relating the aforementioned mechanisms.

## Figures and Tables

**Table 1:** Primer sequences.

Primer Name	Forward	Reverse
p16	5′-CTCGTGCTGATGCTACTGAGGA-3′	5′-GGTCGGCGCAGTTGGGCTCC-3′
p21	5′-AGGTGGACCTGGAGACTCTCAG-3′	5′-TCCTCTTGGAGAAGATCAGCCG-3′
p53	5′-CCTCAGCATCTTATCCGAGTGG-3′	5′-TGGATGGTGGTACAGTCAGAGC-3′
PGC-1α	5′-CCAAAGGATGCGCTCTCGTTCA-3′	5′-CGGTGTCTGTAGTGGCTTGACT-3′
TFAM	5′-GTGGTTTTCATCTGTCTTGGCAAG-3′	5′-TTCCCTCCAACGCTGGGCAATT-3′
p53R2	5′-ACTTCATCTCTCACATCTTAGCCT-3′	5′-AAACAGCGAGCCTCTGGAACCT-3′
